# Dexmedetomidine alleviates hepatic ischaemia‐reperfusion injury via the PI3K/AKT/Nrf2‐NLRP3 pathway

**DOI:** 10.1111/jcmm.16871

**Published:** 2021-10-19

**Authors:** Yan Wu, Gaolin Qiu, Hainie Zhang, Leilei Zhu, Gao Cheng, Yiqiao Wang, Yuanhai Li, Weiwei Wu

**Affiliations:** ^1^ Department of Anesthesiology The First Affiliated Hospital of Anhui University of Chinese Medicine Hefei China; ^2^ Department of Anesthesiology The First Affiliated Hospital of Anhui Medical University Hefei China; ^3^ Department of Anesthesiology The Fourth Affiliated Hospital of Anhui Medical University Hefei China; ^4^ Department of Anesthesiology Anhui NO.2 Provincial People's Hospital Hefei China

**Keywords:** Dexmedetomidine, hepatic ischaemia‐reperfusion injury, hypoxia/reoxygenation cell model, JUND, miR‐494, NLRP3, PI3K/AKT/Nrf2 pathway, pyroptosis

## Abstract

Hepatic ischaemia‐reperfusion (I/R) injury constitutes a tough difficulty in liver surgery. Dexmedetomidine (Dex) plays a protective role in I/R injury. This study investigated protective mechanism of Dex in hepatic I/R injury. The human hepatocyte line L02 received hypoxia/reoxygenation (H/R) treatment to stimulate cell model of hepatic I/R. The levels of pyroptosis proteins and inflammatory factors were detected. Functional rescue experiments were performed to confirm the effects of miR‐494 and JUND on hepatic I/R injury. The levels of JUND, PI3K/p‐PI3K, AKT/p‐AKT, Nrf2, and NLRP3 activation were detected. The rat model of hepatic I/R injury was established to confirm the effect of Dex in vivo. Dex reduced pyroptosis and inflammation in H/R cells. Dex increased miR‐494 expression, and miR‐494 targeted JUND. miR‐494 inhibition or JUND upregulation reversed the protective effect of Dex. Dex repressed NLRP3 inflammasome by activating the PI3K/AKT/Nrf2 pathway. In vivo experiments confirmed the protective effect of Dex on hepatic I/R injury. Overall, Dex repressed NLRP3 inflammasome and alleviated hepatic I/R injury via the miR‐494/JUND/PI3K/AKT/Nrf2 axis.

## 1 INTRODUCTION

Upon restoring blood supply after ischaemia, the liver is vulnerable to further injury, which aggravates the injury caused by ischaemia, termed ischaemia‐reperfusion (I/R) injury.^1^ Hepatic I/R injury commonly occurs during surgical procedures such as hepatectomy and liver transplantation, leading to the graft dysfunction after transplantation.^2^ Multiple factors contribute to hepatic I/R injury, including mitochondrial damage, oxidative stress, intracellular Ca^2+^ overload, activation of apoptotic kinases, cytokines and chemokines generated by kupffer cells and neutrophils.^3^ Some strategies have been developed to alleviate hepatic I/R injury, such as ischaemic preconditioning and postconditioning, and mechanical perfusion; however, these methods are associated with high cost and difficult clinical operation, despite their efficacy in reducing hepatic I/R injury.^4^ Therefore, developing potential pharmacological therapies are critical for improving the prognosis of hepatic I/R injury, especially for those patients receiving surgery with prolonged ischaemia time or marginal liver transplantation.

The restoration of blood flow triggers tissue inflammation and ischaemic injury by activating multiprotein complex named inflammasome.^5^ Inflammasome is implicated in the pathogenesis of hepatic I/R injury, which is accepted as a crucial contributor to hepatocyte injury.^5^, ^6^ After inflammasome activation, the affected tissues undergo apoptosis and another inflammation‐associated cell death, namely pyroptosis.^5^, ^7^ Pyroptosis is a form of lytic programmed cell death initiated by inflammasomes.^8^ Pyroptosis is viewed as a universal and natural immune mechanism in vertebrates, which causes inflammation in bacterial infection and various non‐infectious diseases including types of hepatic injury.^9^ Although there lacks direct evidence of the presence of pyroptosis in hepatic I/R injury, the inflammasome activation in hepatic I/R injury has been elucidated, indicating the involvement of pyroptosis in hepatic I/R injury.^10^ Intriguingly, a previous study has unveiled that inhibition of pyroptosis ameliorates hepatic I/R injury and represses inflammatory reaction.^11^ Activation of some critical survival pathways or inhibition of apoptotic/ pyroptotic pathways using drugs or small molecules contributes to alleviating hepatic I/R injury before or during liver surgery or transplantation.^4^

Dexmedetomidine (Dex), a highly selective α_2_‐adrenergic agonist, bears the potent properties of sedation and analgesia, which is extensively applied in critically ill and anaesthetic patients in clinical.^12^ Accumulating evidences have elucidated that Dex protects lung, cerebrum and liver against I/R injury by suppressing pro‐inflammatory signalling and reducing cell death.^13^, ^14^, ^15^, ^16^, ^17^ For example, Dex exerts protective effect on hepatic I/R injury by repressing inflammatory response and oxidative stress in mice.^18^ Also, the differentially expressed microRNAs (miRNAs) are verified to participate in the mechanisms of Dex action.^19^ miRNA has been accepted as a novel target for treating hepatic I/R injury.^4^ miR‐494 represses hypoxia/reoxygenation (H/R)‐induced cardiomyocyte apoptosis.^20^ miR‐494 attenuates hepatic I/R injury in a rat model.^21^ Overexpression of miR‐494 upregulated HIF‐1α expression under hypoxia and exerts protective effects against hypoxia‐induced apoptosis, which suggests that miR‐494 functions as a therapeutic target for hepatic I/R injury.^22^ Still, whether Dex can protect liver from I/R injury by targeting miR‐494 remains unknown. This study investigated the specific protective mechanism of Dex in hepatic I/R injury, which shall confer novel insights into the management of hepatic I/R injury.

## 2 MATERIALS AND METHODS

### 2.1 Ethics statement

All the animal experiments were implemented on the guide for the care and use of laboratory animals and on minimized animal number and the least pains.

### 2.2 Cell model of H/R

The 293T and human hepatocyte line L02 (Shanghai Institute of Cellular Biology of Chinese Academy of Sciences, Shanghai, China) were identified by STR profiling, and there was no mycoplasma contamination. Cells were cultured in high glucose‐Dulbecco's modified Eagle's medium (4.5 g/L) containing 10% PAN at 37°C with 5% CO_2_. Cells were firstly cultured in HERAcell 150i incubator (Thermo Fisher Scientific, Waltham, MA, USA) at 37°C for 24 h under hypoxia condition (94% N_2_, 5% CO_2_ and 1% O_2_) and then transferred to typical incubator (21% O_2_ and 5% CO_2_) for 4 h for reoxygenation. The cell model induced by H/R was established. After modelling, cells were treated with Dex (20 μM) for 24 h.^23^

### 2.3 Cell transfection and grouping

H/R model cells or 293T cells were transfected with mimic NC, miR‐494 mimic, inhibitor NC, miR‐494 inhibitor, pcDNA3.1‐NC or pcDNA3.1‐JUND (GeneChem, Shanghai, China) (miRNA‐inhibitor 50 nM, miRNA‐mimic 30 nM and pcDNA3.1 10 nM) using LiPofectamine 2000 (11668‐019, Invitrogen, Carlsbad, CA, USA). The subsequent experiments were performed after 48 h. Cells were assigned into blank group (untreated L02 cells), H/R group (H/R‐treated L02 cells), H/R + DEX group (cells were treated with 20 μM Dex for 3 h and then received H/R treatment), H/R + DEX +inhibitor NC group (cells were treated with 20 μM Dex for 3 h and received H/R and then transfected with inhibitor NC), H/R + DEX + inhibitor group (cells were treated with 20 μM Dex for 3 h and received H/R and then transfected with miR‐494 inhibitor), H/R + DEX + pcDNA3.1‐NC group (cells were treated with 20 μM Dex for 3 h and received H/R and then transfected with pcDNA3.1‐NC) and H/R + DEX + pcDNA3.1‐JUND group (cells were treated with 20 μM Dex for 3 h and received H/R and then transfected with pcDNA3.1‐JUND).

### 2.4 Enzyme‐linked immunosorbent assay (ELISA)

The levels of alanine aminotransferase (ALT) (ab234579, Abcam Inc., Cambridge, MA, USA), aspartate aminotransferase (AST) (ab263883, Abcam), IL‐6 (ab178013, Abcam), TNF‐α (ab181421, Abcam), IL‐1β (ab217608, Abcam) and IL‐18 (ab215539, Abcam) was detected using ELISA kits. Briefly, the sample to be tested (containing antibody) was bound to antigen, and then, the labelled enzyme was bound to the complex to form antigen‐antibody‐labelled enzyme complex. The enzyme substrate was added to produce coloured product, and its optical density value was determined by spectrophotometer.

### 2.5 Western blotting

Cells or tissues were treated with RIPA lysis buffer to extract the total protein, and the protein concentration was detected using the bicinchoninic acid kit (Pierce, Waltham, MA, USA). The protein sample was separated by 12% SDS‐PAGE and transferred onto cellulose nitrate membranes (Bio‐Rad Laboratories Inc., Hercules, CA, USA). The membranes were blocked with phosphate‐buffered saline containing 5% skim milk for 2 h and incubated with the primary antibodies at 4°C overnight and then with the secondary antibodies for 2 h. The protein band was developed using enhanced chemiluminescence kit (Thermo Fisher Scientific) and quantified using ImageJ software. The primary antibodies were as followed: anti‐ASC (1:1000, ab151700, Abcam), anti‐Gasdermin‐D (GSDMD)‐N (1:1000, ab215203, Abcam), anti‐caspase‐1 (1:1000, ab207802, Abcam), anti‐PI3K (1:2000, sc‐166365, Santa Cruz Biotechnology, Inc, Santa Cruz, CA, USA), anti‐p‐PI3K (1:5000, ab182651, Abcam), anti‐AKT (1:1,000, sc‐5298, Santa Cruz Biotechnology), anti‐p‐AKT (1:1,000, sc‐293125, Santa Cruz Biotechnology), anti‐Nrf2 (1:1,000, ab89443, ab92946, Abcam), anti‐NLRP3 (1:1000, ab214185, Abcam) and anti‐GAPDH (1:1,000, sc‐47724, Santa Cruz Biotechnology). The secondary antibodies were anti‐mouse IgG (1:2,000, sc‐516102, Santa Cruz Biotechnology) and anti‐rabbit IgG (1:2000, sc‐2357, Santa Cruz Biotechnology).

### 2.6 Reverse transcription quantitative polymerase chain reaction (RT‐qPCR)

Total RNA was extracted from cells or tissues using TRIzol reagent (Takara, Tokyo, Japan), and 500 ng total RNA was reverse transcribed into cDNA using PrimeScript RT Reagent kit (Takara). CFX 96 qPCR system (Bio‐Rad) and SYBR RT‐PCR kit (Takara) were used for RT‐qPCR. The relative expression of miR‐494 and JUND was calculated by 2^−ΔΔCT^ method, with GAPDH and U6 as the internal reference. Each sample was repeated 3 times independently. Primer sequences are shown in Table 1.

**TABLE 1:** Primer sequence for RT‐qPCR

Name of primer	Sequences
hsa‐miR‐494‐F	CGCTGAAACATACACGGGAA
hsa‐miR‐494‐R	CAGTGCAGGGTCCGAGGTAT
rno‐miR‐494‐F	TGGTGATGGGATTTGAAACATACACGGGAAAC
rno‐miR‐494‐R	AGATAGACGG‐TGTCGCTGTTGAAGTCAG
hsa‐JUND‐F	TCCCAGACATGACAGCCATC
hsa‐JUND‐R	TGCTTTGAATCCAAAAACCTTACT
rno‐miR‐JUND‐F	ATGCTGAAGAAAGACGCGCTG
rno‐miR‐JUND‐R	CGCCACCCGCGAAACTGCTCA
hsa‐GAPDH‐F	GACCTGACCTGCCGTCTA
hsa‐GAPDH‐R	AGGAGTGGGTGTCGCTGT
rno‐GAPDH‐F	GGAGTCTACTGGCGTCTTCAC
rno‐GAPDH‐R	ATGAGCCCTTCCACGATGC
hsa‐U6‐F	CTCGCTTCGGCAGCACA
hsa‐U6‐R	AACGCTTCACGAATTTGCGT
rno‐U6‐F	GCTTCGGCAGCACATATACTAAAAT
rno‐U6‐R	GCTTCACGAATTTGCGTGTCAT

### 2.7 Animal model

Sprague Dawley (SD) rats of specific pathogen‐free (SPF) grade (weighing 190–210 g and aged 9–10 weeks) were provided by Shantou University Medical College [SCXK (Guangdong) 2017‐0017]. The rats were reared in SPF animal room at 60% humidity and 22–24°C. Food and water were provided ad libitum. The rats were maintained in a 12 h light/dark cycle.

antagomiR‐494 and antagomiR‐NC were purchased from GenScript (Nanjing, Jiangsu, China). The rats were administered via tail vein 3 days before modelling (consecutive 3 days; 40 μg/g).

The rats were anaesthetized by intraperitoneal injection of 1% pentobarbital (50 mg/kg) and fixed on the heating pad in supine position. The incision was made along the midline of the upper abdomen. The hepatic pedicle was clamped with a non‐invasive microvascular clamp, and hepatic I/R was induced 60 min later. Dex was administered intravenously at the beginning of the operation at a loading dose of 3 μg/kg and then at a dose of 3 μg/kg/min for the next 2 h.^24^ The rats in the sham group received all operations except clamping the hepatic pedicle. After abdominal suture, the rats were allowed to recover with free access to food and water. After 24 h of reperfusion, rats were euthanized by intraperitoneal injection of 1% pentobarbital (800 mg/kg). The blood was extracted from left ventricle by a syringe, centrifuged at 2000 g and 4°C for 15 min and stored at −80°C. The rat liver tissues were collected and fixed in 4% paraformaldehyde. The rats were assigned into sham group (rats received all operations except clamping the hepatic pedicle), I/R group (rats received I/R treatment), I/R + DEX group (rats received I/R treatment and intravenous injection of Dex), I/R + DEX + antagomiR‐NC group (after antagomiR‐NC transfection, rats received I/R treatment and intravenous injection of Dex) and I/R + DEX + antagomiR‐494 group (after antagomiR‐494 transfection, rats received I/R treatment and intravenous injection of Dex). Each group had 12 rats, among which 6 rats were used for tissue section staining and 6 rats were used for protein or RNA extraction and detection.

### 2.8 Haematoxylin and eosin (HE) staining

The liver tissues fixed in 4% paraformaldehyde for 24 h were embedded in paraffin, sliced continuously with the thickness of 5 μm, baked at 58°C for 18 h and dewaxed with xylene and hydrated. The sections were incubated in haematoxylin solution for 15 min and stained with eosin solution for 15 s. The sections were dehydrated, cleared and sealed, followed by observation under optical microscope.

### 2.9 Suzuki score

Suzuki score was used to evaluate liver tissue injury in rats, and the Suzuki score criteria are shown in Table 2 below.

**TABLE 2:** Suzuki's criteria of hepatic I/R injury

Score	Congestion (%)	Vacuolization (%)	Necrosis (%)
0	None	None	None
1	Minimal (10)	Minimal (10)	Single cell necrosis
2	Mild (11–30)	Mild (11–30)	Mild (<30)
3	Moderate (31–60)	Moderate (31–60)	Moderate (>60)
4	Severe (>60)	Severe (>60)	Severe (>60)

### 2.10 Immunohistochemical staining

The liver tissue sections were treated with 0.01 mol/L citric acid buffer (95°C 5 min × 2 times) for antigen retrieval and incubated with 3% H_2_O_2_ at room temperature for 15 min and with the primary antibody anti‐NLRP3 (1:200, ab214185, Abcam) at 4°C overnight. Afterwards, the sections were cultured with the secondary antibody anti‐rabbit IgG (1:2000, ab150077, Abcam) for 30 min, stained with 3‐diaminobenzidine (Sigma‐Aldrich, Merck KGaA, Darmstadt, Germany), washed with running water and then counterstained with haematoxylin.

### 2.11 Statistical analysis

Data were analysed and introduced using SPSS 21.0 (IBM Corp., Armonk, NY, USA). Data are expressed as mean ± standard deviation. Kolmogorov‐Smirnov test was used to test the normal distribution. The *t* test was adopted for comparison between two groups. One‐way analysis of variance (ANOVA) was employed for the comparisons among multiple groups, following Tukey's multiple comparisons test. The *p* value was obtained from a two‐tailed test and the *p* < 0.05 meant the statistically significance.

## 3 RESULTS

### 3.1 Dex exerted protective effects on hepatic I/R injury by suppressing pyroptosis

**FIGURE 1:**
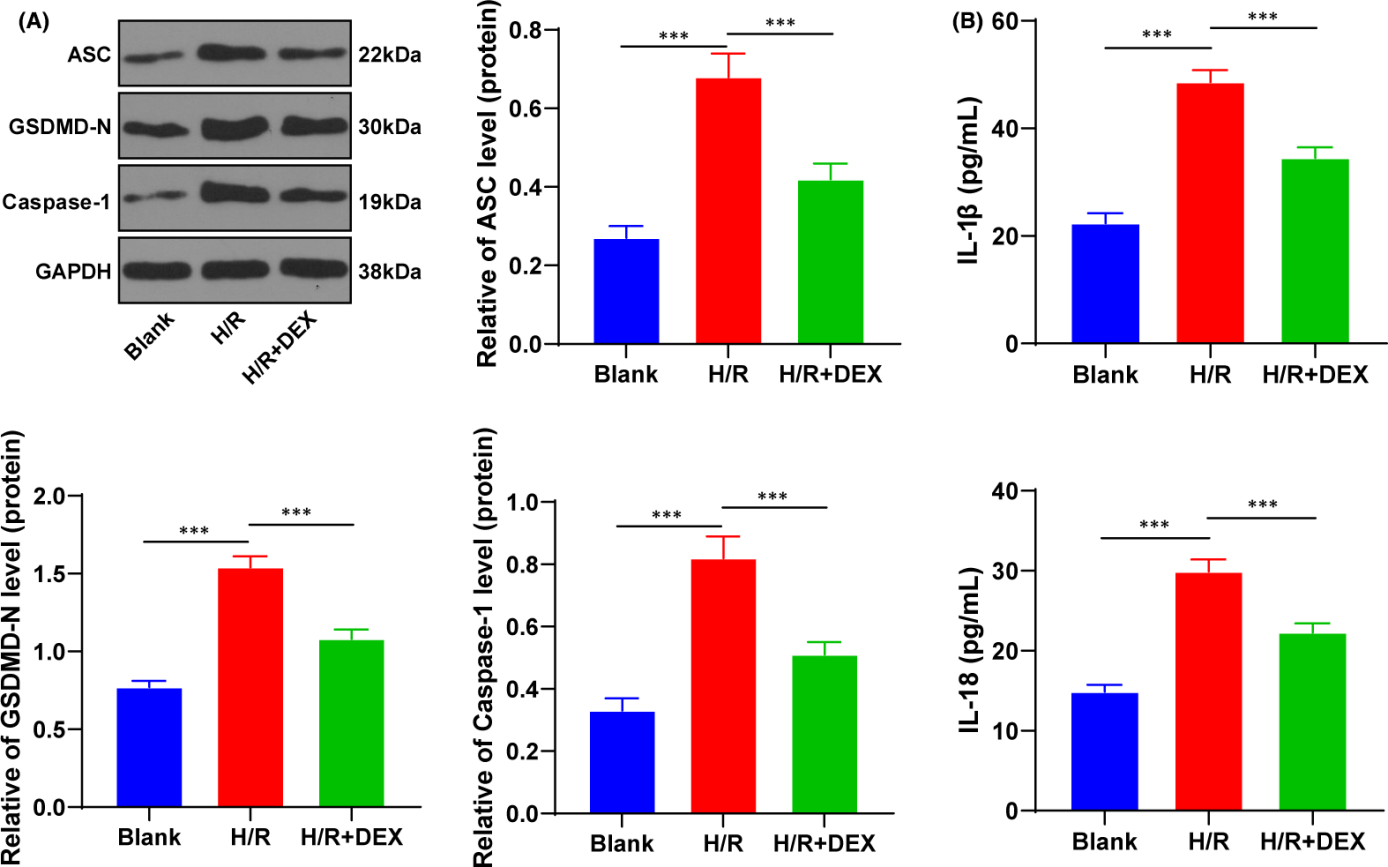
Dex exerted protective effects by suppressing pyroptosis. The H/R cell model was treated with Dex. A, The pyroptotic proteins ASC, GSDMD‐N and caspase‐1 in H/R cells were detected using Western blotting. B, The contents of IL‐1β and IL‐18 were detected using ELISA. The cell experiment was repeated 3 times. Data were presented as mean ± standard deviation and analysed using one‐way ANOVA, followed by Tukey's multiple comparison test, ^***^ *p* < 0.001

To observe the effect of Dex on cell model, we simulated hepatic I/R injury in human hepatocyte line L02 through H/R treatment. Pyroptosis, also known as inflammatory necrosis, is characterized by cell swelling until cell membrane rupture, leading to the release of cellular contents and activation of strong inflammatory response.^25^ We speculated that Dex played a protective role by suppressing pyroptosis. The protein levels of ASC, GSDMD‐N and caspase‐1, and the contents of IL‐1β and IL‐18 were detected using Western blotting and ELISA (Figure 1A,B). The results revealed that the levels of ASC, GSDMD‐N, IL‐1β and IL‐18 in the H/R group were notably higher than those in the blank group, while Dex treatment decreased the levels of ASC, GSDMD‐N, IL‐1β and IL‐18 (all *p* < 0.001). Briefly, Dex protected the cell model of I/R injury by suppressing pyroptosis.

### 3.2 Dex protected H/R cells through miR‐494

**FIGURE 2:**
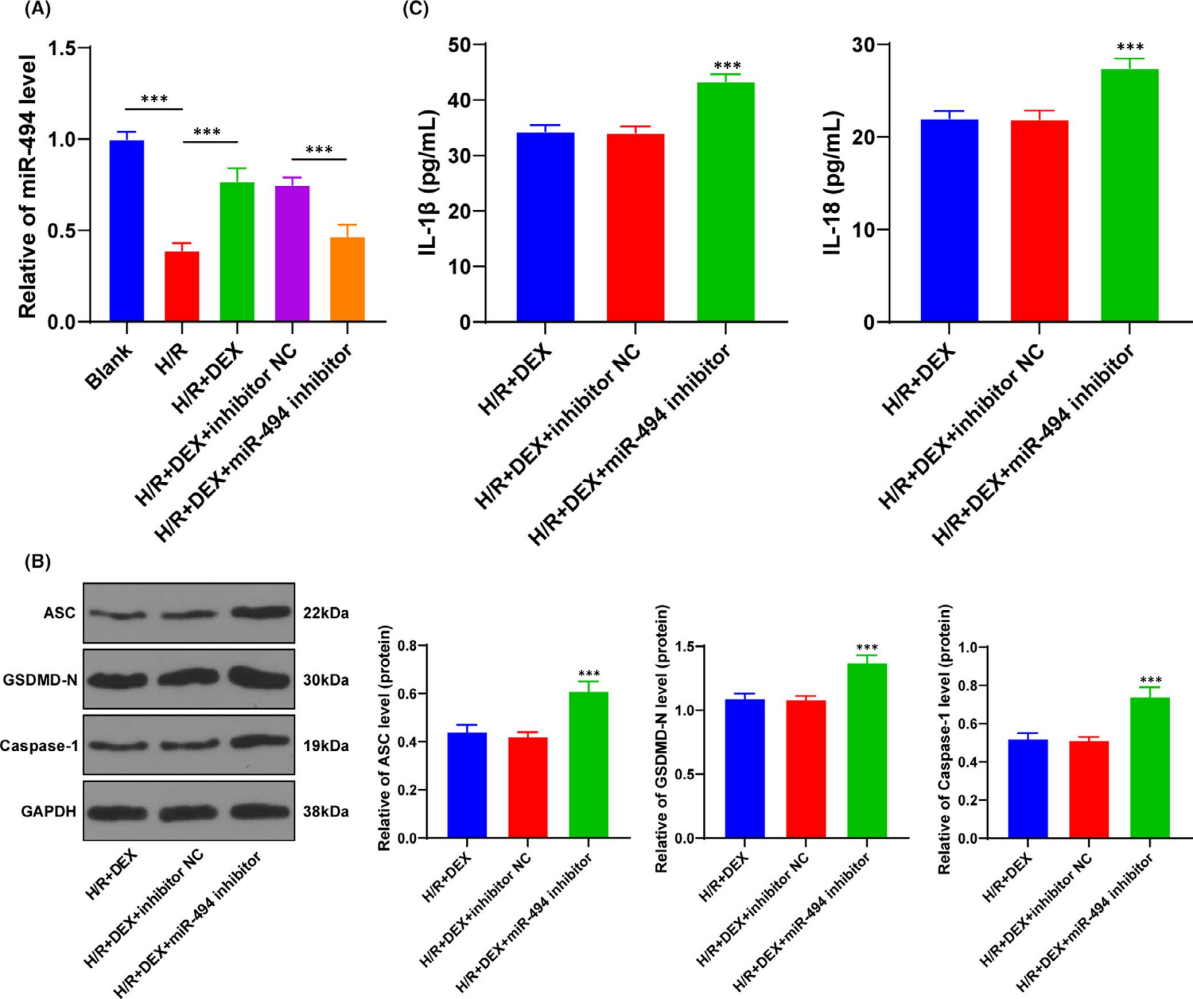
Dex protected H/R cells through upregulating miR‐494. The expression of miR‐494 in each group and the effect of Dex on H/R cells after transfection of miR‐494 inhibitor was observed. A, miR‐494 expression in each group was detected using RT‐qPCR. B, The pyroptotic proteins were detected using Western blotting. C, The contents of inflammatory factors were detected using ELISA. The cell experiment was repeated 3 times. Data were presented as mean ± standard deviation and analysed using one‐way ANOVA, followed by Tukey's multiple comparison test, ^***^ *p* < 0.001

miRNA plays a vital role in hepatic I/R injury and may become a novel tool to diagnose and monitor hepatic I/R injury.^26^ miR‐494 can play a protective role in liver I/R injury.^21^ Therefore, we speculated that Dex protected H/R‐treated cells through miR‐494. miR‐494 expression of L02 cells in the blank group, H/R group and H/R + DEX group (Figure 2A) was detected using RT‐qPCR. The results demonstrated that H/R treatment downregulated miR‐494 expression of L02 cells (*p* < 0.001), while Dex inhibited the downregulation of miR‐494 to a certain extent (*p* < 0.001). Subsequently, we transfected miR‐494 inhibitor into H/R cells to further verify the role of miR‐494 (Figure 2A). The levels of ASC, GSDMD‐N and caspase‐1, and the contents of IL‐1β and IL‐18 were detected (Figure 2B,C): miR‐494 inhibitor reversed the protective effect of Dex on H/R cell pyroptosis and attenuated the inhibition of Dex on inflammatory factors. Briefly, Dex attenuated H/R cell pyroptosis by upregulating miR‐494.

### 3.3 miR‐494 targeted JUND

**FIGURE 3:**
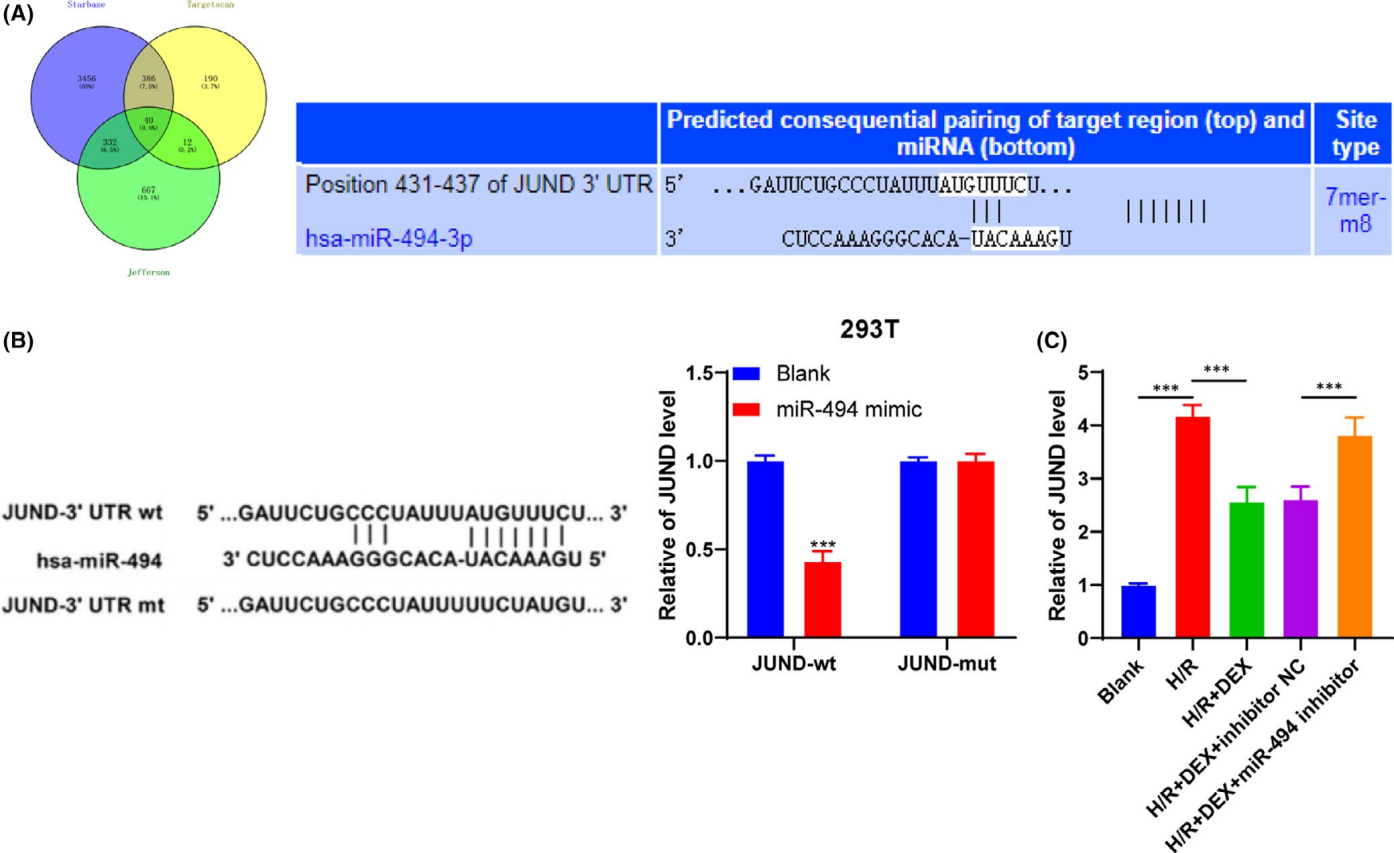
miR‐494 targeted JUND. After the targeting relationship between miR‐494 and JUND was confirmed in HEK293T cells, it was verified in cell experiment groups. A, The binding site of miR‐494 and JUND was predicted through the target gene prediction websites. B, The binding relationship between miR‐494 and JUND was verified using dual‐luciferase reporter assay. C, JUND expression in each group was detected using RT‐qPCR. The cell experiment was repeated 3 times. Data were presented as mean ± standard deviation. Data in panel C were analysed using one‐way ANOVA, and data in panel B were analysed using unpaired *t* test, followed by Tukey's multiple comparison test, ^***^ *p* < 0.001

To investigate the potential mechanism of miR‐494 in H/R cells, we predicted the downstream targets of miR‐494 through Starbase (http://starbase.sysu.edu.cn/), TargetScan (http://www.targetscan.org/vert_72/) and Jefferson (https://cm.jefferson.edu/rna22/Precomputed/). After taking the intersection, we focused on JUND (Figure 3A), because accumulating evidences have unveiled the role of JUND in liver injury.^27^, ^28^ Next, the dual‐luciferase reporter assay in HEK293T cells verified the binding relationship between miR‐494 and JUND (Figure 3B). Subsequently, the relative expression of JUND in the above experimental groups was detected using RT‐qPCR (Figure 3C), and the results exhibited that miR‐494 suppressed JUND expression. Briefly, miR‐494 targeted JUND.

### 3.4 Upregulation of JUND reversed the protective effect of Dex on H/R cells

**FIGURE 4:**
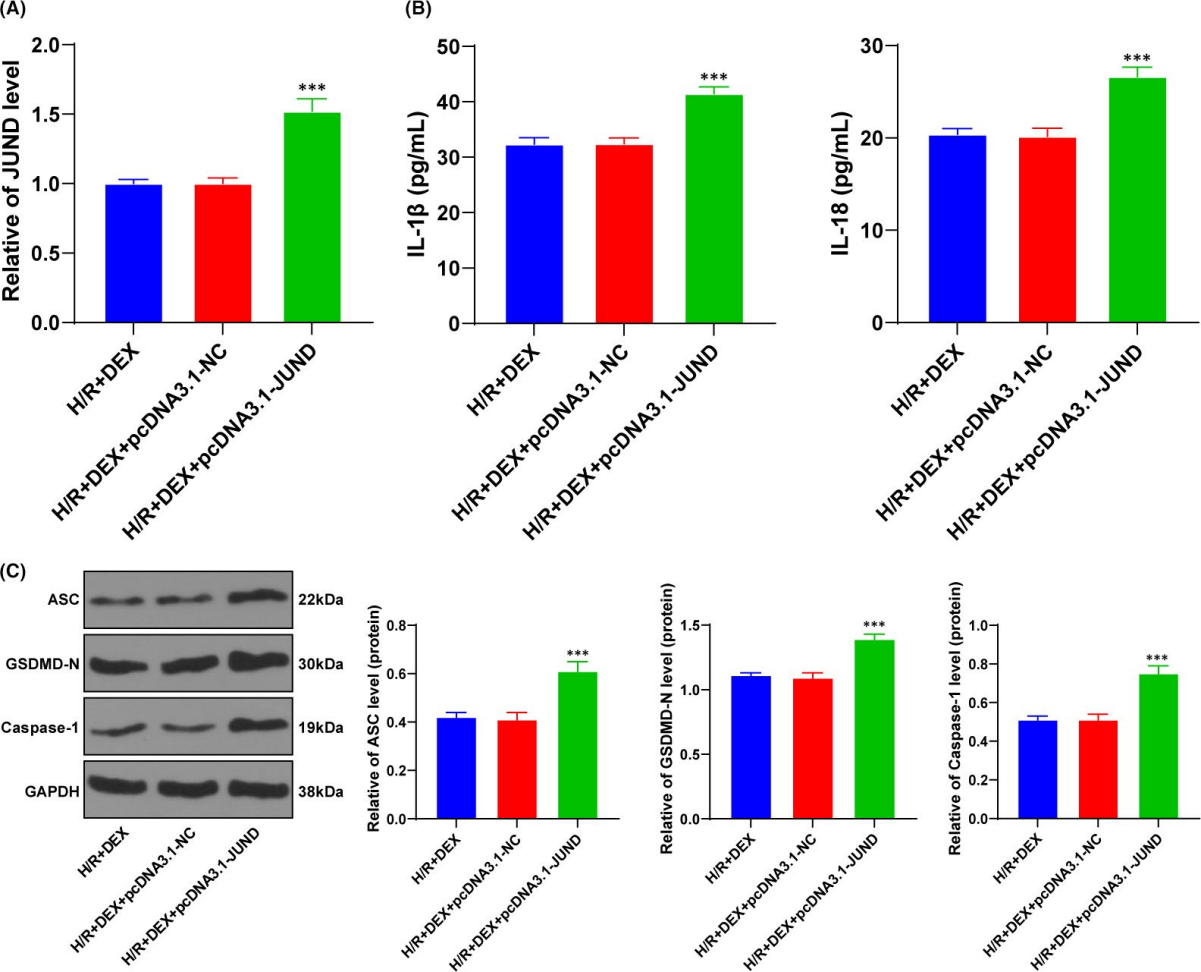
Upregulation of JUND reversed the protective effect of Dex on H/R cells. The effects of Dex on H/R cell pyroptosis and inflammatory factors were observed after JUND was overexpressed in the model cells. A, JUND expression was detected using RT‐qPCR. B, The pyroptotic proteins were detected using Western blotting. C, The contents of inflammatory factors were detected using ELISA. The cell experiment was repeated 3 times. Data were presented as mean ± standard deviation and analysed using one‐way ANOVA, followed by Tukey's multiple comparison test, ^***^ *p* < 0.001

To verify the regulatory mechanism of the above targeting relationship in Dex, we transfected pcDNA3.1‐JUND into cells. RT‐qPCR confirmed the transfection efficiency (Figure 4A). The release of pyroptosis proteins and inflammatory factors were detected using Western blotting and ELISA (Figure 4B,C): overexpression of JUND partially reversed the effect of Dex on H/R cells. It was suggested that upregulation of JUND reversed the protective effect of Dex on H/R cells.

### 3.5 Dex repressed NLRP3 inflammasome by activating the PI3K/AKT/Nrf2 pathway

**FIGURE 5:**
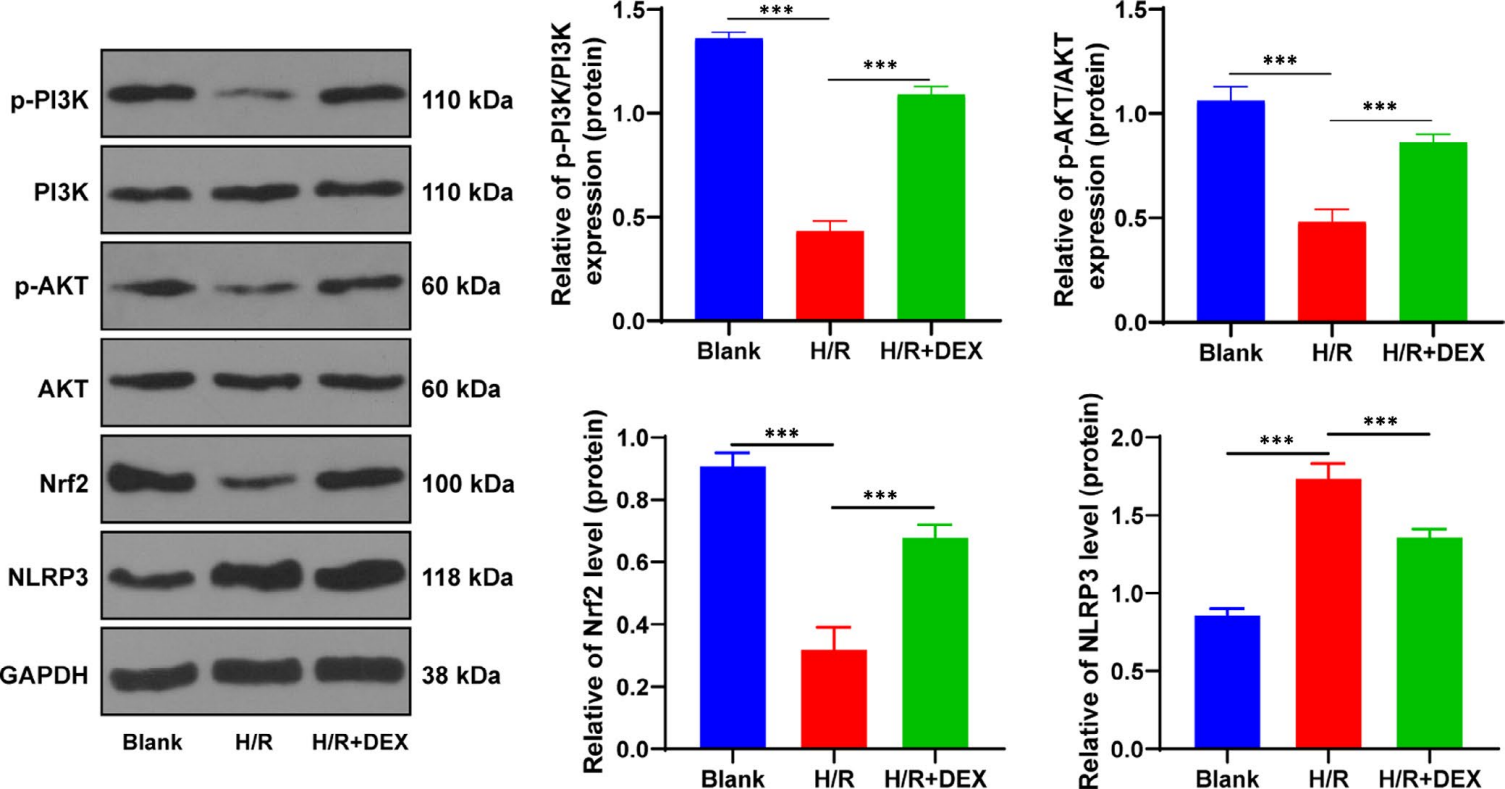
Dex repressed NLRP3 inflammasome by activating the PI3K/AKT/Nrf2 pathway. The effect of Dex on PI3K/AKT/Nrf2 pathway and the effect of Nrf2 on NLRP3 inflammasome were observed by detecting the expression of Nrf2 and phosphorylation of PI3K and AKT in each group. A, Nrf2, PI3K, p‐PI3K, AKT, p‐AKT and NLRP3 levels were detected using Western blotting. The cell experiment was repeated 3 times. Data were presented as mean ± standard deviation and analysed using one‐way ANOVA, followed by Tukey's multiple comparison test, ^***^ *p* < 0.001

**FIGURE 6:**
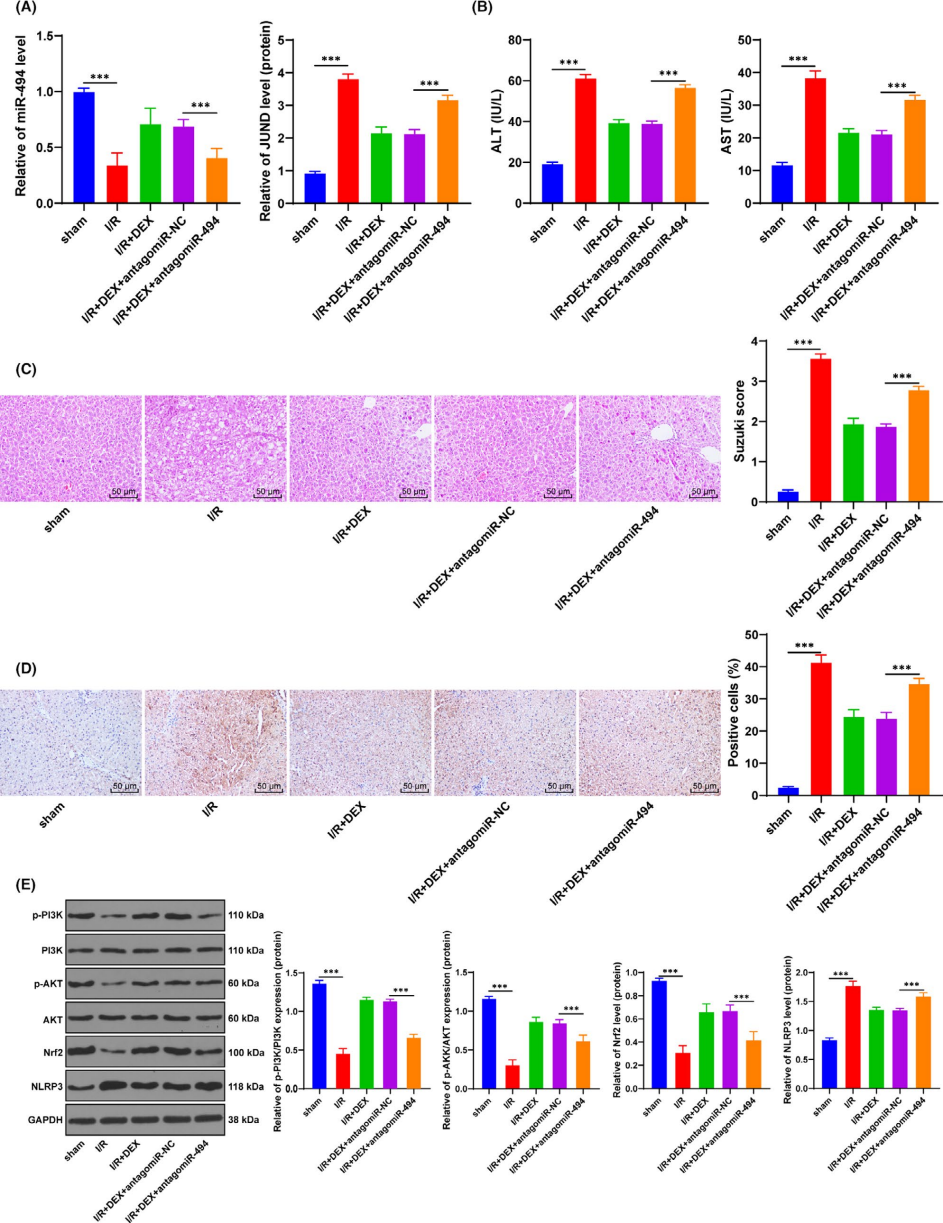
Dex protected hepatic I/R injury in vivo. The rat model of hepatic I/R was established. A, miR‐494 and JUND expression was detected using RT‐qPCR. B, The levels of ALT and AST in serum of rats were detected using ELISA. C, The rat liver sections were stained with HE staining. D, The rat liver sections were stained with immunohistochemical staining. E, The changes of JUND, p‐AKT, AKT, Nrf2 and NLRP3 were detected using Western blotting. *N* = 6. Data were presented as mean ± standard deviation and analysed using one‐way ANOVA, followed by Tukey's multiple comparison test, ^***^ *p* < 0.001

PI3K/AKT/Nrf2 pathway is implicated in the protection of liver injury.^29^, ^30^ Therefore, we detected the levels of PI3K/p‐PI3K, AKT/p‐AKT and Nrf2 in each group (Figure 5A). The results revealed that H/R treatment inhibited the phosphorylation of PI3K and AKT and the expression of Nrf2, while Dex reversed these trends to some extent (*p* < 0.001), thus promoting the activation of the PI3K/AKT/Nrf2 pathway. Nrf2 activation restrains the NLRP3 inflammasome activation,^31^ which was also observed by Western blotting (Figure 6A). Combined with the changes of inflammatory factors (ASC, GSDMD‐N, caspase‐1, IL‐1β and IL‐18) and the expressions of miR‐494 and JUND, it was indicated that Dex repressed the activation of the PI3K/AKT/Nrf2 pathway via miR‐494/JUND, thus suppressing NLRP3 inflammasome.

### 3.6 Dex protected hepatic I/R injury in vivo

To verify the above cell experiment results, we established the rat model of hepatic I/R injury. miR‐494 mRNA expression and JUND protein level in the rats were detected (Figure 6A). The levels of ALT and AST in the serum of rats in the I/R group were higher than those in the sham group, while Dex reduced the levels of ALT and AST (Figure 6B) (*p* < 0.001). The rat liver sections were observed by HE staining (Figure 6C): Dex treatment notably decreased Suzuki score of hepatic injury^32^ (*p* < 0.001); NLRP3 expression was detected by immunohistochemical staining (Figure 6D): NLRP3 was significantly enhanced in the I/R group, while Dex decreased the positive expression of NLRP3. Inhibition of miR‐494 reversed the protective effect of Dex (*p* < 0.001). JUND expression in vivo was detected using RT‐qPCR (Figure 6A). The phosphorylation of PI3K/AKT/Nrf2 pathway‐related proteins and the NLRP3 protein level were detected using Western blotting (Figure 6E). The results were consistent with the above cell experiment results (*p* < 0.001). Taken together, Dex exerted protective effect on hepatic I/R injury in rats.

## 4 DISCUSSION

The liver is highly dependent on oxygen supply and vulnerable to hypoxia; and the adverse consequences of hepatic I/R injury remain a tough issue in clinical practice.^33^ Dex is an anaesthetic adjuvant that can reduce inflammatory reaction during the perioperative period, suggesting that it may become a novel therapeutic approach to alleviate I/R injury.^34^ The pathogenesis of hepatic I/R injury includes the occurrence of oxidative stress and the release of pro‐inflammatory factors, and Ma XG, et al. found that Dex reduced H/R‐induced elevation of ROS and MDA levels.^35^ Meanwhile, Zhou H, et al. found that Dex pretreatment can promote the activation of macrophage M2 and inhibit the activation of hepatic inflammatory innate immunity in a PPARγ/STAT3 dependent manner.^36^ In this study, we found the Dex regulates the activation of NLRP3 inflammasomes by regulating miR‐494/JUND, which further confirmed that Dex protects hepatic I/R by inhibiting inflammation in the liver.

Hepatic I/R induces inflammation and oxidative stress, resulting in the injury of liver and distant organs.^37^ Dex alleviates inflammatory response and shows protective effect in various animal models of I/R injury.^13^ The liver enzymes ALT and AST are commonly utilized to evaluate hepatic injury; and α‐GST serves as a sensitive marker of hepatic I/R injury.^38^ Our results demonstrated that Dex treatment notably reduced the levels of α‐GST, IL‐6, TNF‐α, ALT and AST in serum, suggesting that Dex exerted protective effects on hepatic I/R injury clinically. Dex mitigates hepatic I/R injury, and its protective mechanism may be concerned with reducing biochemical factors AST and ALT and inflammatory cytokines and enhancing Bcl‐2, thereby attenuating inflammatory response and suppressing apoptosis in mice.^18^, ^24^ Our study confirmed the protective role of Dex in hepatic I/R injury.

To observe the effect of Dex on cell model, we simulated hepatic I/R injury in human liver cell line L02 through H/R treatment. Inflammasome activation has been recently described as a promising therapeutic target of hepatic I/R injury.^39^ Typical inflammasomes, including NLRP3, recruit caspase‐1 under a series of microbial stimulation and endogenous risk signals, and then caspase‐1 induces a form of lytic programmed cell death called pyroptosis.^40^ It is well established that inflammasome activation is responsible for pyroptosis.^41^ Pyroptosis is featured by the activation of caspase‐1, which eventually results in the cleavage of GSDMD and the secretion of IL‐1β and IL‐18.^37^ GSDMD is a genetic substrate of inflammatory caspases, which is implicated in pyroptosis and IL‐1β release.^25^ ASC represents a scaffold protein that is essential to recruit effector enzyme pro‐caspase‐1 into NLRP3 inflammasome.^42^ Accumulating evidences have unveiled the critical role of pyroptosis in the pathological process of hepatic I/R, and repression of pyroptosis contributes to alleviating hepatic I/R injury.^25^, ^43^, ^44^ Accordingly, we found that Dex treatment decreased the levels of ASC, GSDMD‐N, IL‐1β and IL‐18, indicating that Dex protected the cell model of I/R injury by suppressing pyroptosis. Dex is demonstrated to suppress caspase‐1 activation and reduce pyroptosis, thus protecting cells from HMGB1‐induced cell injury.^45^ Consistently, Dex ameliorates myocardial I/R injury in rats and represses H/R‐induced pyroptosis in cardiomyocytes by reducing miR‐29b.^46^ These results indicated that Dex protected the cell model of I/R injury by suppressing pyroptosis.

Then, we investigated the molecular mechanism of Dex protecting liver from I/R injury. Aberrant miRNA expression is implicated in the pathogenesis of I/R injury, because they modulate the cell participants and humoral factors related to I/R injury.^26^ *miR‐494* is differentially expressed in cerebral I/R injury in rats^47^ and shows protective effect on myocardial I/R injury.^48^ We speculated that Dex protected H/R cells through miR‐494. As our results indicated, H/R treatment downregulated miR‐494 expression, while Dex inhibited the downregulation of miR‐494 to a certain extent. Additionally, miR‐494 inhibitor reversed the effect of Dex on H/R cell pyroptosis and the release of inflammatory factors. Similarly, miR‐494 protects rats against hepatic I/R injury by targeting its downstream gene PTEN.^21^ Taken together, Dex attenuated H/R cell pyroptosis by upregulating miR‐494.

Subsequently, we predicted the downstream target genes of miR‐494 through websites. JUND is a member of the activator protein‐1 family of transcription factors, which regulates inflammation by targeting IL‐1β synthesis and macrophage activation.^49^ Importantly, the differential expression of JUND has been reported in hepatic I/R injury.^27^ This study confirmed that miR‐494 targeted JUND. Functional rescue experiment revealed that overexpression of JUND partially reversed the protective effect of Dex on H/R cells. In consistency, JUND overexpression can increase the infarct size following myocardial I/R injury.^50^

miR‐494 attenuates hepatic I/R injury in a rat model by activating the PI3K/AKT pathway.^21^ PI3K/AKT signalling plays a vital role in hepatic I/R injury by suppressing pro‐apoptotic signals and inflammation.^51^ Activating the PI3K/AKT signalling is conducive to the remission of hepatic I/R injury.^51^, ^52^, ^53^ Nrf2 activation facilitates cell protection in a PI3K‐dependent manner by reducing oxidative stress, hepatic inflammation and hepatocyte necrosis/apoptosis.^54^, ^55^ Nrf2 activation is a potent intervention that can protect hepatic I/R injury during and after surgery.^56^ In this study, H/R treatment inhibited the phosphorylation of PI3K and AKT and the level of Nrf2, while Dex reversed these trends and activated the signalling pathway. The inhibition of oxidative stress and apoptosis observed in Dex‐treated mice with I/R injury may be attributed to the enhanced Nrf2.^18^ Excessive activation of NLRP3 facilitates myocardial, cerebral and hepatic I/R injury.^57^ The crosstalk between Nrf2 and inflammasome has been unveiled, and Nrf2 activation represses NLRP3 inflammasome and inflammation,^31^, ^58^ which is consistent with the results of Western blot in our study. NLRP3 silencing exerts protective effects on hepatic I/R injury in mice mainly by downregulating caspase‐1 activation and reducing IL‐1β and IL‐18 secretion.^59^ Briefly, Dex repressed NLRP3 inflammasome by activating the PI3K/AKT/Nrf2 pathway. Moreover, in vivo experiments verified that Dex reduced the positive expression of NLRP3, and Dex had a protective effect on hepatic I/R injury in rats.

To sum up, Dex upregulated miR‐494 expression to inhibit JUND and activated the PI3K/AKT/Nrf2 pathway, thereby repressing NLRP3 inflammasome and alleviating hepatic I/R injury. This study simply revealed that Dex inhibited inflammation through miR‐494 and its target gene to protect liver from I/R injury, but the specific mechanism of Nrf2 in NLRP3 inflammasome failed to be deeply studied. Nrf2 activation is tightly related to its entry and exit from the nucleus, and the regulation mechanism of out‐ and inside nucleus of Nrf2 in NLRP3 remains to be studied. In the future, we will further study on the mechanism of Nrf2 in regulating NLRP3, which may provide novel insights into the clinical treatment of hepatic I/R injury.

## CONFLICT OF INTEREST

The authors declare that they have no conflicts of interest.

## AUTHOR CONTRIBUTIONS

**Yan Wu:** Conceptualization (equal); data curation (equal); methodology (equal); project administration (equal); writing–review and editing (equal). **Gaolin Qiu:** Conceptualization (equal); data curation (equal); writing–original draft (equal); writing–review and editing (equal). **Hainie Zhang:** Formal analysis (equal); methodology (equal). **Leilei Zhu:** Methodology (equal); supervision (equal). **Gao Cheng:** Investigation (equal); writing–review and editing (equal). **Yiqiao Wang:** Formal analysis (equal); writing–original draft (equal). **Yuanhai Li:** Data curation (equal); formal analysis (equal). **Weiwei Wu:** Data curation (equal); formal analysis (equal); writing–original draft (equal); writing–review and editing (equal).

## Data Availability

All the data generated or analysed during this study are included in this published article.
